# Exploring the Clinical Applications and Implications of EMDR Therapy for PTSD in Forensic Settings: A Systematic Review

**DOI:** 10.1192/j.eurpsy.2025.1522

**Published:** 2025-08-26

**Authors:** S. Prasad, G. S. Gill, S. Gunturu

**Affiliations:** 1 Psychiatry, BronxCare Health System, New York; 2 Psychiatry, Case Western Reserve University, Cleveland, United States

## Abstract

**Introduction:**

There is a scarcity of research on the application of EMDR in addressing PTSD within correctional facilities, presenting varied results due to the difficulties associated with prolonged imprisonment. Prisoners encounter persistent mental health conditions such as depression, trauma, PTSD, and substance use disorders, contributing to maladaptive behaviors within the aggressive and psychologically taxing prison environment. Racial tensions and the pressure to conceal emotions further complicate matters, affecting daily life within the prison setting.

**Objectives:**

To conduct a systematic review, following PRISMA guidelines, to assess the efficacy and evidence supporting the use of Eye Movement Desensitization and Reprocessing (EMDR) therapy for Posttraumatic Stress Disorder (PTSD) within forensic settings, in order to address the limited research available and provide insights into the outcomes and challenges related to treating PTSD in incarcerated individuals.

**Methods:**

A review of 11 studies meeting the inclusion criteria was conducted. The study involved 156 incarcerated participants with a median age of 36.9 years, examining gender-based differences in reported trauma experiences and evaluating the effectiveness of EMDR therapy for PTSD in forensic environments. Primary assessment tools included PCL-C and CAPS scores for pre- and post-therapy evaluations. The timing of the initial EMDR session post-incarceration and symptomatic improvements over sessions were analyzed.

**Results:**

Analysis of the study revealed significant improvements in symptoms over an average of six EMDR sessions, with no reported adverse events during therapy. The study highlighted the challenges and outcomes of implementing EMDR therapy for PTSD in correctional settings, shedding light on the effectiveness of the treatment among incarcerated individuals.

**Conclusions:**

Despite positive initial results, it is vital to approach the interpretation of the benefits of EMDR therapy with caution, given factors such as the absence of standardized clinical trials, variability in reported outcomes, and the potential for study bias. Recognizing the need for additional investigation, further exploration is warranted to understand the lasting effects of EMDR therapy on recidivism rates, taking into account variables like the nature of the offense, length of incarceration, and frequency of reoffending.

**Disclosure of Interest:**

None Declared

